# A Case of Coexistence of Esophageal Squamous Cell Carcinoma and Adenocarcinoma

**DOI:** 10.7759/cureus.81794

**Published:** 2025-04-06

**Authors:** Margaret L Munz, Lorenzo K Sampson

**Affiliations:** 1 Surgery, Edward Via College of Osteopathic Medicine, Spartanburg, USA; 2 Surgery, Aiken Regional Medical Center, Aiken, USA

**Keywords:** carcinoma esophagus, esophagus and gastric cancer surgery, gi oncology, upper endoscopy, upper gastrointestinal surgery

## Abstract

Esophageal malignancy is a prevalent cancer both in the United States and globally. In the United States, adenocarcinoma is a particularly common esophageal malignancy, likely secondary to gastroesophageal reflux disease (GERD) and Barrett’s esophagus. Conversely, squamous cell carcinoma (SCC) is the most common histology globally, especially prevalent in Asian countries, in what is known as the esophageal cancer belt. SCC and adenocarcinoma of the esophagus have clinical, oncologic, and histologic differences suggesting the need for tailored therapeutic approaches. In some cases, patients with concurrent risk factors for both malignancies can present with a collision tumor or adjacent tumors with two histologic subtypes within a single mass, and this warrants a unique treatment strategy. A case such as this one, involving a patient presenting with simultaneous poorly differentiated SCC as well as high-grade adenocarcinoma in the background of tubulovillous adenoma-like change, presents a unique clinical course and a possible example of a collision tumor. Furthermore, the differing prognostic indicators for both squamous and adenocarcinoma of the esophagus can highlight the importance of individualized treatment plans to improve both patient satisfaction and quality of life.

## Introduction

Esophageal malignancy is the eighth most common cancer in the world [1]. Esophageal cancer affects about four in 100,000 in the United States, most commonly men over the age of 60 [1]. Adenocarcinoma is the most common esophageal malignancy in the United States, with an increasing incidence resulting from gastroesophageal reflux disease (GERD) and Barrett’s esophagus [2], while squamous cell carcinoma (SCC) is the most common globally, and is especially prevalent in Asian countries, in the “esophageal cancer belt” [2]. SCC is associated with lower socioeconomic status, nicotine use, and tobacco and alcohol abuse, and is often seen with comorbid cirrhosis [3]. SCC is most commonly located in the proximal 75% of the esophagus [3]. Adenocarcinoma is associated with higher socioeconomic status, cardiovascular risk factors, and obesity, and is often located in the distal 25% of the esophagus [3]. Other risk factors for esophageal cancer include human papillomavirus (HPV) infection, occupational exposures such as cleaning solvents, or preexisting esophageal conditions such as achalasia [1]. Adenocarcinoma results from a dysplasia progression through Barrett's esophagus or intestinal metaplasia of the distal esophagus [3]. SCC is associated with earlier lymphatic spread and a worse prognosis [3]. 

Often esophageal cancer remains asymptomatic and is diagnosed after it has spread, with only 25% of patients diagnosed prior to metastasis [1]. Symptoms of esophageal carcinoma include dysphagia due to obstruction of the mass, increased heartburn from esophageal reflux, weight loss secondary to disease or decreased oral intake, increased coughing or hemoptysis, voice changes, and odynophagia [1]. 

The workup for esophageal malignancy often includes esophagogastroduodenoscopy (EGD) with biopsy. Other initial options include barium swallow, EGD with ultrasound, and CT and PET scans for diagnosis of metastasis [1]. Therapeutic modalities include surgery on occasion for localized disease, chemotherapy and radiation, endoscopic laser therapy, photodynamic therapy, and immunomodulators [1]. Options for surgery can include esophagectomy for advanced disease; other options include endoscopic mucosal resection or endoscopic laser therapy [1]. 

It is important to consider the differing histologic origins of two distinct forms of esophageal cancer because the disease pathogenesis differs based on the inciting factors. This results in differing presentations of the two cancers. SCC and adenocarcinoma of the esophagus have clinical, oncologic, and histologic differences and literature suggests this warrants different therapeutic approaches [4]. 

## Case presentation

This patient, a 61-year-old African-American male, presented to the office after a referral from a primary care provider for anemia. The patient complained of a several-week history of "crampy" abdominal pain and postprandial epigastric “burning sensation.” The patient also complained of dysphagia, which improved when lying on his left side. The patient was recently started on proton pump inhibitors, with minimal relief. The patient had a history of chronic meloxicam use as well as a smoking history but had successfully quit over 30 days prior. The patient reported social alcohol use, about one to two drinks per month (beer). The patient was up to date on his screening colonoscopies, with no abnormal findings. The patient denied any other surgical or endoscopic procedures prior to presenting. The patient was taking appropriate medications for his chronic conditions. These include hypertension, hyperlipidemia, spondylosis (pain treated with meloxicam), and he recently began pantoprazole for the treatment of GERD. 

The physical exam revealed an alert-appearing male in no cardiopulmonary distress. The patient did not have any palpable lymphadenopathy. His abdominal exam revealed a non-tender, non-distended abdomen with normal bowel sounds. The patient had slight tenderness to palpation over the epigastric region. 

The patient's labs were essentially normal and non-contributory. The patient had very mild microcytic anemia on 10/4/23 with a hemoglobin of 11 g/dL (13.5-17.5 g/dL) and a mean corpuscular volume of 77 fL (80-100 fL). These initial labs were ordered by the primary care physician. All other values were within normal limits.

**Figure 1:**
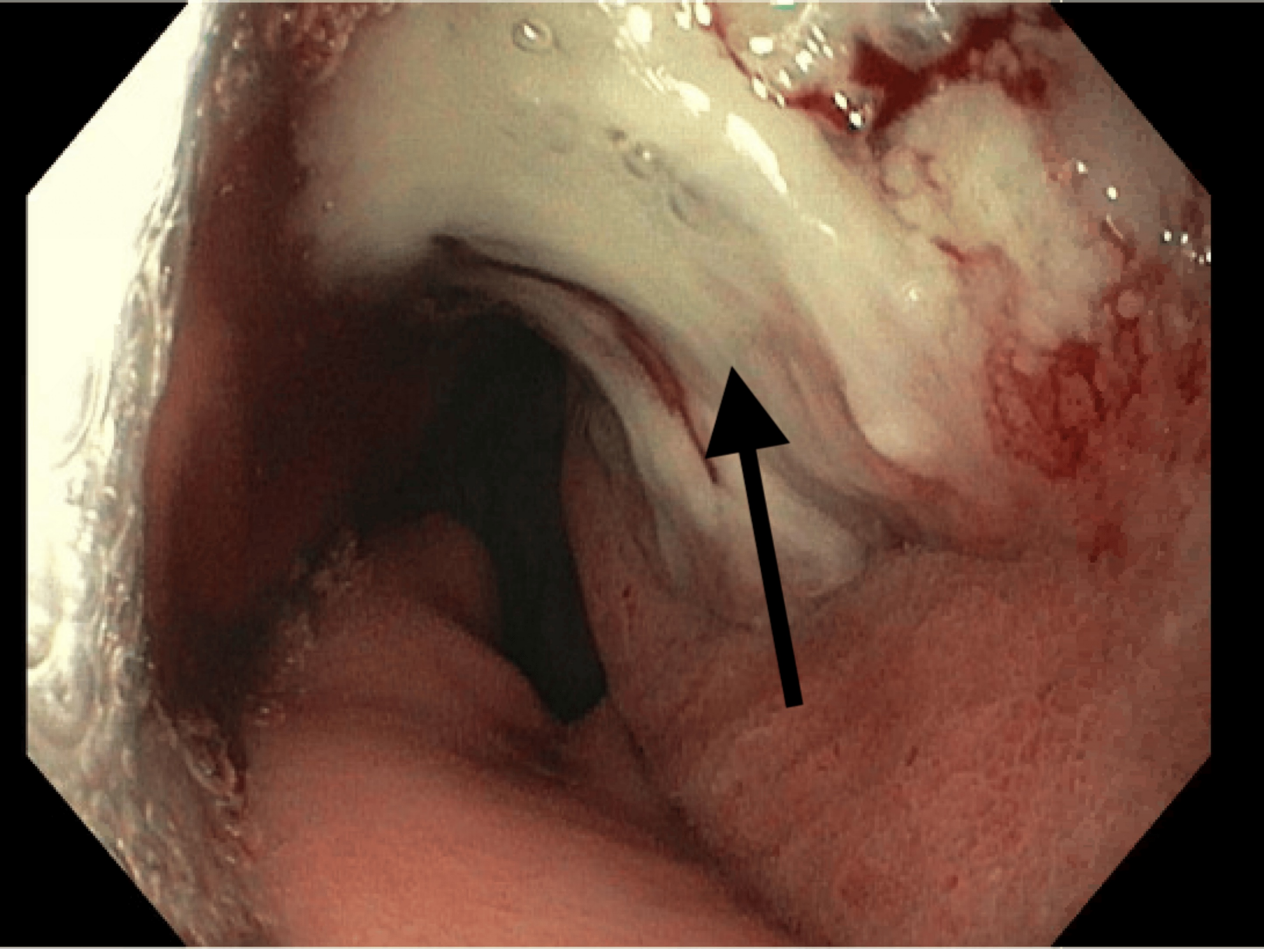
Gross image from upper endoscopy showing a partially obstructing esophageal mass near the gastroesophageal junction

**Figure 2:**
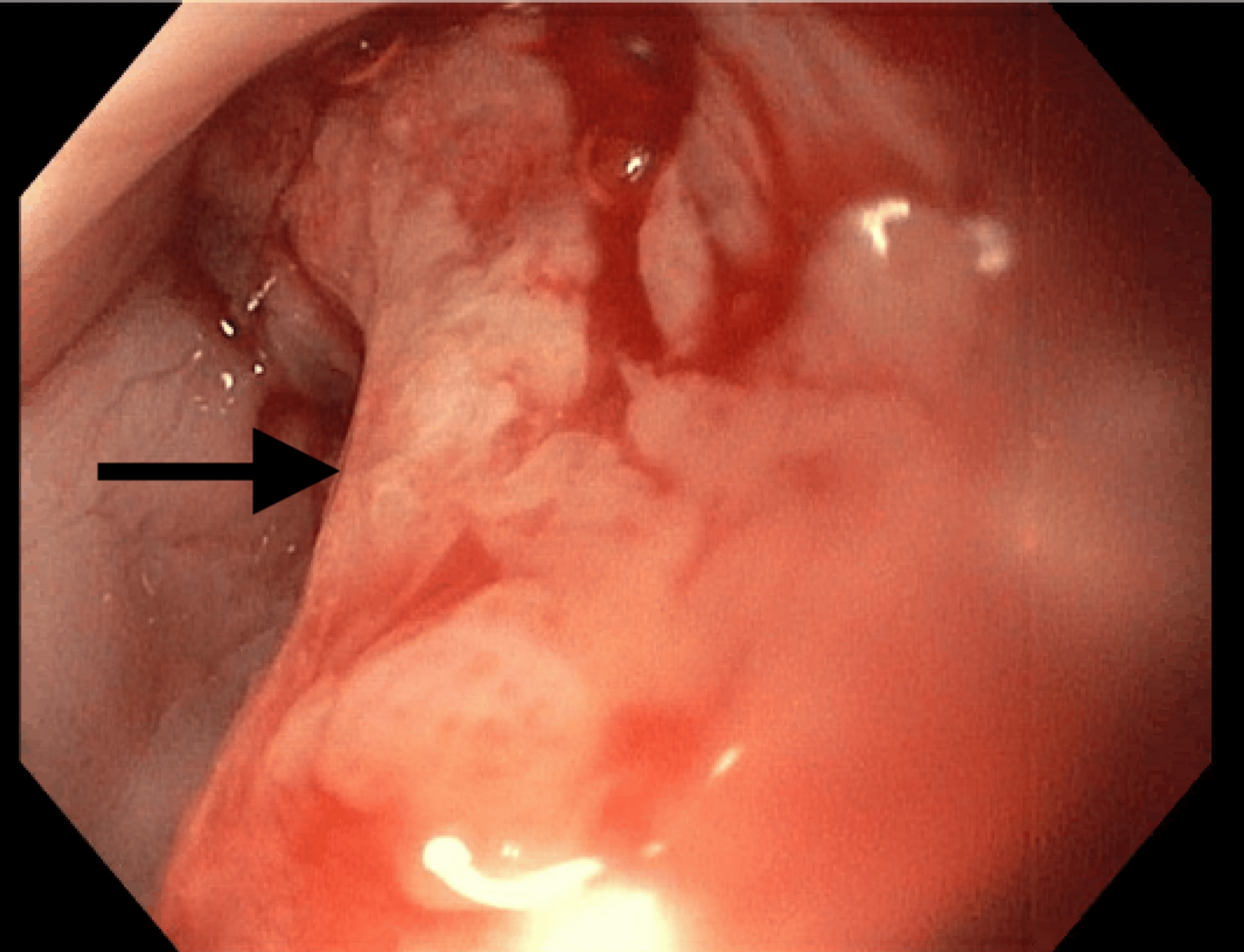
Grossly friable esophageal mass located in the distal third of the esophagus

**Figure 3:**
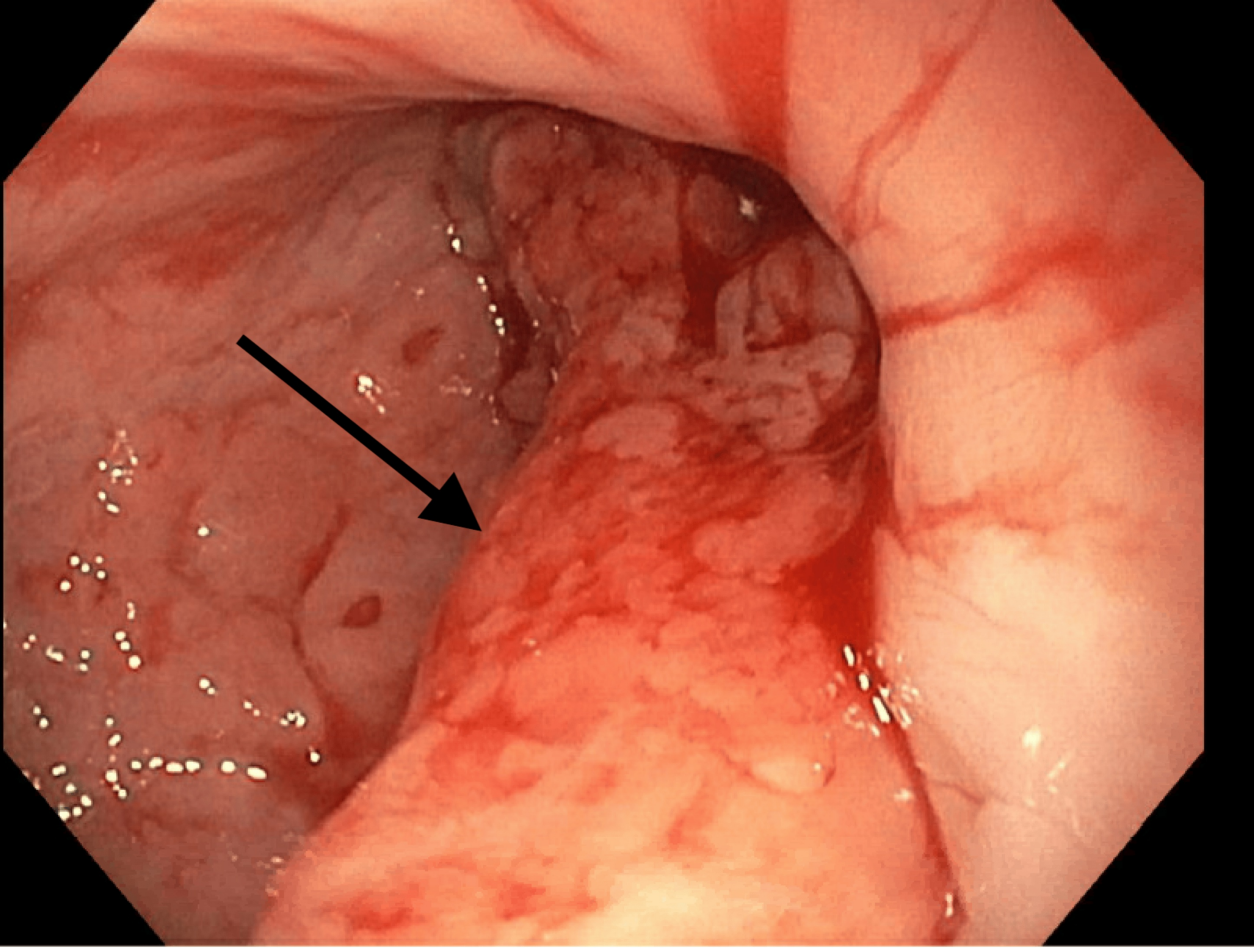
Additional view of partially obstructing mass in the distal esophagus

The patient underwent EGD with a diagnostic biopsy. As seen on endoscopy, a tumor was measured to be between 33 cm and 41 cm from the central incisors (Figure 1). The mass was friable and extended into the gastric cardia; inflammation and erythema can be observed from gross images (Figure 2 and Figure 3). Biopsy confirmed invasive poorly differentiated SCC with focal spindled features and invasive high-grade adenocarcinoma with mucinous features and mild signet ring characteristics, arising in the background of tubulovillous adenoma-like changes with high-grade dysplasia and scattered goblet cells in the lower third of the esophagus and the gastric cardia. Further staging through biopsy was not possible due to the biopsy method and the absence of a fully resected tumor mass. The patient underwent a PET scan, which showed focal intense radiotracer activity in the distal esophagus, local regional metastasis to the subcarinal lymph node, and distant metastatic diffuse activity in the central mesentery with ascites.

**Figure 4:**
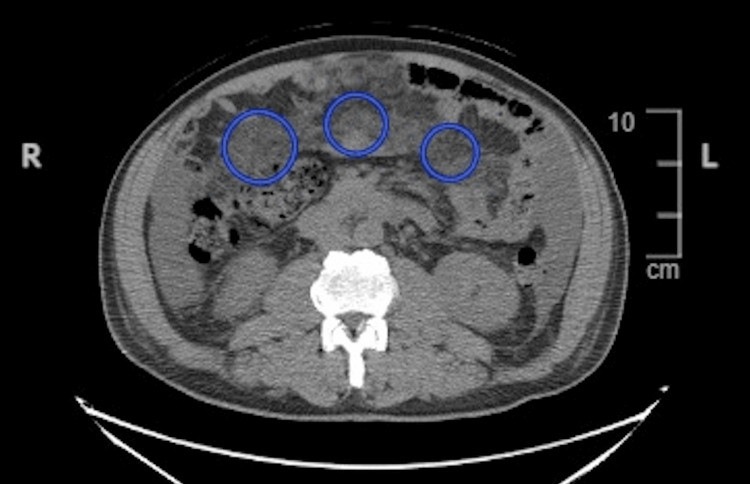
Abdominal scan demonstrating diffuse metastatic activity to the central mesentery (blue circles) as well as associated ascites

**Figure 5:**
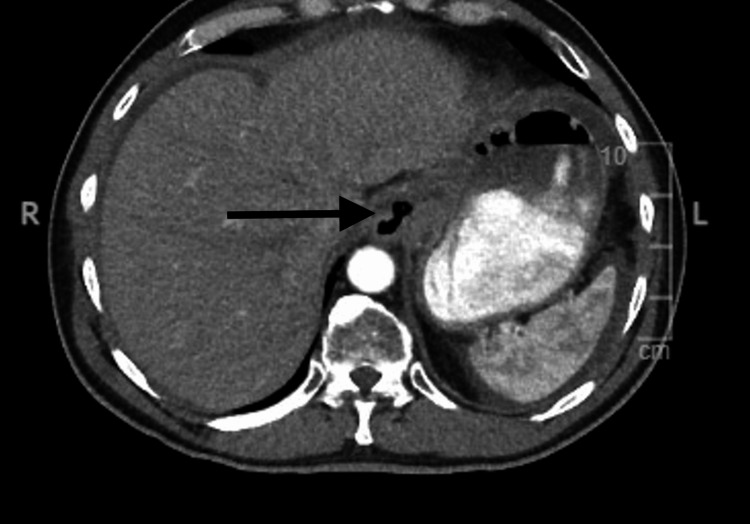
Abdominal CT scan showing thickening of the distal esophagus (black arrow) consistent with malignant disease

Additionally, abdominal and chest CT scans were performed for staging, which revealed mesenteric thickening of the greater omentum, circumferential thickening of the distal esophagus and gastric cardia, and lymphadenopathy (Figure 4 and Figure 5). The imaging studies were concerning for metastatic disease and suggested that resection would not be curative. The patient underwent chemoradiation therapy and was referred to oncology for radiation and chemotherapy treatments. The patient was recommended to receive FOLFOX (fluorouracil [5-FU], oxaliplatin, and leucovorin) and OPDIVO (nivolumab, a PD-L1 inhibitor, Bristol-Myers Squibb) [5-7]. The patient is scheduled for an additional PET scan at the end of his next chemotherapy cycle to evaluate surgical candidacy. Currently, the patient is not experiencing obstructive symptoms or dysphagia and therefore does not require palliative surgery at this time. However, palliative surgery may be considered in the future if disease progression interferes with adequate nutrition. The patient is being treated on an outpatient basis and is in stable condition. He underwent port-a-cath placement and is compliant with chemoradiation treatment, which he is tolerating well. The patient is scheduled for follow-up in the surgery clinic after the completion of his chemotherapy cycle and a repeat PET scan to assess surgical candidacy.

Thus, a case such as this, where a patient presents with simultaneous poorly differentiated SCC and high-grade adenocarcinoma in the background of tubulovillous adenoma-like changes, presents a unique clinical course. Collision tumors result in a rare and distinctive presentation that may warrant specific treatment [2]. The patient was referred for chemotherapy, as well as CT and PET scans for surveillance of malignant disease. The PET scan revealed increased activity at the gastroesophageal junction. This case presents a rare instance of simultaneous esophageal malignancies and underscores the importance of better understanding disease progression, given the poor prognosis of these cases.

## Discussion

The International Agency for Research on Cancer predicts that esophageal cancer will increase by 50% from 2020 to 2040 [8]. Therefore, considering the clinical, histologic, and oncologic differences between the various forms of esophageal cancer is critical in tailoring specific treatment approaches [3,9-11,12-14]. Additionally, the roles of chemotherapy, targeted therapy, surgery, and radiation are determined on a patient-specific basis. While both SCC and adenocarcinoma have unique risk and prognostic factors, it is crucial to recognize that many patients have risk factors for both [8]. In this case, our patient had both a smoking history and chronic GERD. 

Collision tumors result in a unique and rare presentation that may warrant specific treatment [2]. Traditionally, SCC is associated with lower socioeconomic status, nicotine, and alcohol use, while adenocarcinoma is associated with high socioeconomic status, obesity, and cardiovascular risk factors [8,10-12,15,16]. Additionally, the surgical approach differs based on tumor morphology and location. SCC is typically located in the proximal third of the esophagus, and surgery usually requires a subtotal esophagectomy with cervical anastomosis, while adenocarcinoma in the distal third of the esophagus can be resected with an intrathoracic anastomosis (Ivor-Lewis procedure) [8]. ​

There are various treatments that can be considered when addressing esophageal cancer. Surgery is an option to treat local disease or for palliative care if the tumor burden is too great and the patient has significant dysphagia, discomfort, or malnutrition. Esophagectomy will remove the diseased portion while a stent is rarely done but could be considered for palliative measures [4]. Radiation, both internal and external, can be used to decrease disease burden. Chemotherapy, as seen in this case, or neoadjuvant chemotherapy can be used in conjunction with surgery and radiation or alone [4]. Other less commonly used treatment options include laser therapy, electrocoagulation, immunotherapy, and targeted therapy [4]. 

One potential limitation of the analysis of this patient case is that it was not possible to obtain the histology slides for inclusion in this publication. The pathologist reported that due to the manner in which biopsies were taken and placed in the same container, further grading was not possible at this time. The pathologist states he does not believe this tumor is "adenosquamous" as that represents a different disease pathology. In his opinion, without resection of the entire tumor or thorough examination, the tumor likely arose secondary to Barrett's esophagus while a simultaneous SCC originated adjacent to it; the tumors appeared as one mass or a collision tumor. It was impossible to determine if the original origin was in the distal esophagus or gastric cardia and/or in reference to the Z-line. It is possible that this patient's smoking history and meloxicam use contributed to his GERD and secondary Barrett's esophagus; it is now more widely recognized in the literature that SCC and adenocarcinoma have concurrent risk factors [3,17]. Total resection was not indicated for this patient’s treatment but it is recognized that this would be the best way to definitively grade and diagnose the histologic subtype of the tumor. 

Given the predicted increased incidence over the next decade, as well as the significant disease mortality (46% five-year survival in those diagnosed with early-stage esophageal carcinoma), it is critical that policies and protocols are adjusted to best prevent disease burden [5,1]. Alongside primary prevention, such as avoiding key risk behaviors including smoking and obesity, early screening in high-risk populations to diagnose esophageal cancer before it metastasizes [5] is essential to reduce disease burden and mortality. According to the 2024 NCCN guidelines, upper endoscopy at regular intervals is recommended for nearly all stages of esophageal cancer and this can be accompanied by other tests [9,18]. 

## Conclusions

This case presents a rare instance of simultaneous esophageal malignancies and highlights the importance of better understanding disease progression, given the poor prognosis of these cases. Both forms of malignancy most commonly present as severe, stage IV disease. Many authors suggest that the pathologic, histologic, and clinical differences between SCC and adenocarcinoma warrant an individualized clinical approach. Additionally, with a predicted increase in the incidence of esophageal cancer in general, it is crucial to integrate primary prevention and raise awareness of risk factors for at-risk patients. Early screening and diagnosis are also key in the treatment of these rapidly metastatic cancers. Continued research and studies are needed to better understand and appreciate the molecular nuances of the pathology and treatment of esophageal cancer.
